# High-Dose Intravenous Immunoglobulins in the Treatment of Severe Acute Viral Pneumonia: The Known Mechanisms and Clinical Effects

**DOI:** 10.3389/fimmu.2020.01660

**Published:** 2020-07-14

**Authors:** Xiaosheng Liu, Wei Cao, Taisheng Li

**Affiliations:** ^1^Tsinghua-Peking Center for Life Sciences, School of Medicine, Tsinghua University, Beijing, China; ^2^Department of Infectious Diseases, Peking Union Medical College Hospital, Peking Union Medical College and Chinese Academy of Medical Sciences, Beijing, China

**Keywords:** intravenous immunoglobulin, SARS-CoV-2, COVID-19, viral pneumonia, mechanism of action, therapeutic inference

## Abstract

The current outbreak of viral pneumonia, caused by novel coronavirus SARS-CoV-2, is the focus of worldwide attention. The WHO declared the COVID-19 outbreak a pandemic event on Mar 12, 2020, and the number of confirmed cases is still on the rise worldwide. While most infected individuals only experience mild symptoms or may even be asymptomatic, some patients rapidly progress to severe acute respiratory failure with substantial mortality, making it imperative to develop an efficient treatment for severe SARS-CoV-2 pneumonia alongside supportive care. So far, the optimal treatment strategy for severe COVID-19 remains unknown. Intravenous immunoglobulin (IVIg) is a blood product pooled from healthy donors with high concentrations of immunoglobulin G (IgG) and has been used in patients with autoimmune and inflammatory diseases for more than 30 years. In this review, we aim to highlight the known mechanisms of immunomodulatory effects of high-dose IVIg therapy, the immunopathological hypothesis of viral pneumonia, and the clinical evidence of IVIg therapy in viral pneumonia. We then make cautious therapeutic inferences about high-dose IVIg therapy in treating severe COVID-19. These inferences may provide relevant and useful insights in order to aid treatment for COVID-19.

## Introduction

Recently, the outbreak of a febrile respiratory disease caused by a novel beta-coronavirus, designated SARS-CoV-2, has become the focus of worldwide attention. The event was declared a Pandemic by the WHO on Mar 12, 2020, and the number of confirmed cases is still climbing worldwide. Typical clinical features of COVID-19 include fever, respiratory symptoms such as dry cough and shortness of breath, and fatigue (1). While the majority of infected individuals experience only mild symptoms or may even be asymptomatic, about 10 to 20% of patients rapidly progress to severe conditions with acute respiratory distress syndrome (ARDS), which can result in shock, sepsis, and multiple organ dysfunction (1, 2). In addition to supportive treatment, several therapeutic approaches have been proposed, such as antiviral therapy, immunomodulators, blood products, and traditional Chinese medicine (TCM) (3, 4). However, the optimal treatment strategy for severe COVID-19 remains debated (5–11). To date, it remains an urgent requirement to identify the most effective treatment for severe and critically ill COVID-19 patients, but a conclusion has not yet been drawn.

The severity of respiratory viral infections depends on a fine balance between the virulence of the pathogen and the inflammatory response of the host's immune system (12). Although an effective immune response is essential to eliminate viral pathogens, clinical observations and experimental data indicate that an excessive host immune response—rather than direct viral damage—primarily account for the pathological injury and clinical deterioration of COVID-19 (12). Inflammatory cytokine storms and lymphopenia are the signature features of patients with severe COVID-19, indicating an intense systemic inflammatory response during SARS-CoV-2 infection (13). Specific adjuvant immunomodulatory treatments have been considered for severe viral infections, but some of these were associated with a worse prognosis. Therefore, it is vital to select appropriate immunomodulators in COVID-19 patients and to carefully assess their benefits and risks (14, 15).

Based on the previous clinical experience in China, it was proposed that early initiation of high-dose intravenous immunoglobulins (IVIg) and low-molecular-weight heparin might be effective in improving the prognosis of severe and critically ill COVID-19 patients (16, 17). The national diagnosis and treatment protocol for COVID-19 (Trial Version 7) and recommendations from the Peking Union Medical College Hospital recommend appropriate application of IVIg therapy in severe and critical COVID-19 cases (4, 18). However, the specific molecular mechanisms and clinical effectiveness of IVIg treatment remain unclear. Here, we review the current knowledge of immunomodulatory mechanisms of high-dose IVIg and discuss its potential role in treating severe viral infection. Moreover, we summarize the clinical efficacy of IVIg therapy in treating severe viral pneumonias such as that caused by SARS, MERS, influenza, and RSV disease, and its current application in COVID-19. A thorough understanding of the immunomodulatory mechanisms of IVIg therapy is crucial for the timing and dosing of early intervention and may provide much-needed insights into the immunomodulatory treatment of severe and critically ill COVID-19 patients.

## Immunomodulatory Mechanisms of High-Dose IVIG Therapy

IVIg is a blood preparation isolated and concentrated from healthy donors consisting of over 95% of IgG and trace amounts of IgA or IgM. IVIg has been used in clinical practice for many years. Different doses of IVIg serve diverse medical indications, and the clinical efficacy of IVIg differs with dosage (19). At low and moderate doses, IVIg can be administered as substitutional therapy for primary or acquired immunodeficiencies in order to improve plasma IgG concentrations and provide passive immunity. Apart from this, IVIg can also be used as immunomodulatory therapy at high doses to treat autoimmune or inflammatory disease such as immune thrombocytopenia (ITP) or Kawasaki disease, and has several off-label indications including for hematologic, dermatologic, neuromuscular, and rheumatologic disorders (20) (Table 1).

**Table 1:** Major indications of IVIg therapy.

**IVIg as replacement therapy**	
Primary antibody immunodeficiency^*^ Ataxia telangiectasia Common variable immunodeficiency syndrome Severe combined immunodeficiency Wiskott Aldrich syndrome X-linked agammaglobulinemia X-linked hyper-IgM syndrome	**Secondary antibody immunodeficiency** B-cell chronic lymphocytic leukemia^*^ Transplantation Multiple myeloma Pediatric HIV infection Drugs induced
**IVIg as immunomodulatory therapy**	
**Hematologic disorders** Immune thrombocytopenic purpura^*^ Auto-immune hemolytic anemia Auto-immune neutropenia anemia HIV-associated thrombocytopenia Neonatal alloimmune thrombocytopenia Parvovirus B19-associated aplasia **Dermatologic disorders** Atopic dermatitis Bullous pemphigoid Blistering diseases Dermatomyositis Epidermolysis bullosa acquisita Immune urticaria Kawasaki syndrome^*^ Mucous-membrane (cicatricial)pemphigoid Pemphigus vulgaris Pyoderma gangrenosum Scleromyxoedema Stevens-Johnson syndrome Toxic epidermal necrolysis	**Neuromuscular disorders** Birdshot retinopathy Chronic inflammatory demyelinating polyneuropathy^*^ Guillain Barre syndrome Lambert–Eaton myasthenic syndrome Multifocal motor neuropathy^*^ Myasthenia gravis Opsoclonus–myoclonus Paraneoplastic syndromes Persistent epilepsy disorder Polyradiculoneuropathy Relapsing-remitting multiple sclerosis Stiff person syndrome **Ophthalmologic disorders** Autoimmune uveitis Birdshot retinochoroidopathy Mucous membrane pemphigoid **Rheumatologic disorders** Vasculitis Systemic lupus erythematosis Streptococcal toxic shock syndrome

* *FDA approved indications*.

**Figure 1:**
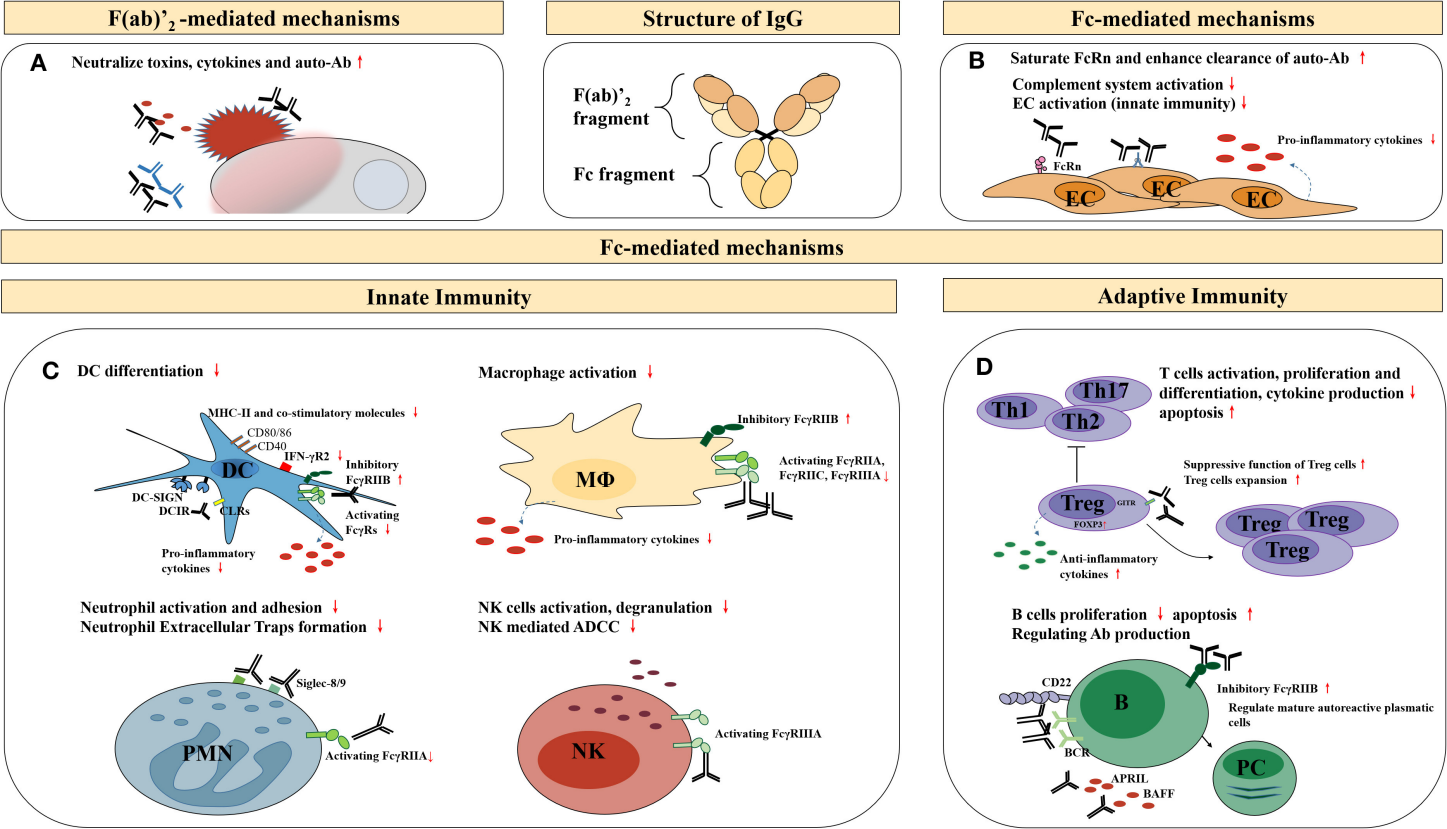
Potential anti-inflammatory, immunomodulatory mechanisms of high-dose IVIg therapy. EC, endothelial cells; DC, dendritic cells; MΦ, macrophages; PMN, granular leukocyte; NK, natural killer cells; ADCC, antibody-dependent cell-mediated cytotoxicity; B, B cells; PC, plasma cells; BCR, B-cell receptors; APRIL, a proliferation-inducing ligand; BAFF, B-cell activating factor of the TNF family. **(A)** Neutralization of pathogenic antigens through the F(ab)′2-mediated mechanisms; **(B)** The immunomodulatory effects on endothelial cells through the Fc-mediated mechanisms; **(C)** The immunomodulatory effects on other innate immune cells through the Fc-mediated mechanisms; **(D)** The immunomodulatory effects on adaptive immune cells through the Fc-mediated mechanisms.

The underlying molecular mechanisms of IVIg therapy effectiveness are conferred by IgG and its two functional domains, namely the F(ab)′_2_ fragment (the dimeric antigen-binding fragment), which is responsible for specific antigen binding, and the Fc fragment (crystallizable fragment), responsible for Fc receptor (FcR) and complement binding. Several hypotheses have been proposed to explain the immunomodulatory mechanisms of high-dose IVIg, including the F(ab)′_2_-mediated and the Fc-mediated mechanisms (Figure 1).

### F(ab)′_2_-Mediated Mechanisms

#### Neutralization of Pathogenic Antigens, Including Microorganisms or Toxins

The well-known microbial antigen-specific binding properties of the IgG-F(ab)′_2_ fragment provide the basis for passive immunity that allows for immediate protection against microbes in immunodeficient patients. *In vitro* and preclinical experiments confirmed that IVIg contains multivalent pathogen-specific neutralizing IgG antibodies against common opportunistic pathogens and toxins (e.g., *Enterococcus* spp., *Haemophilus influenzae* type b, *Streptococcus pyogenes, Staphylococcus aureus*, cytomegalovirus, and Shiga toxins) (21–26). Variance in pathogen-specific antibody titer and neutralizing activity is usually observed among various IVIg preparations, and is most likely due to differences in the geographical regions where plasma samples were collected, reflecting differences in pathogen exposure (27–29).

#### Neutralization of Endogenous Antigens Including Inflammatory Cytokines, Chemokines, Complement Fragments, and Apoptosis-Related Molecules

In addition to microbial binding, a wide range of endogenous antigen-specific IgG are also present in IVIg preparations. These endogenous antigens may include: inflammatory cytokines (e.g., IL-1α, IFN-α2a, and GM-CSF), chemokines (e.g., CCR5), apoptosis-related molecules (e.g., Siglec-8, Siglec-9, CD95/Fas, BAFF, and APRIL), complement fragments (C3a and C5a), and anti-idiotypic antibodies (e.g., anti-amyloid β antibodies and anti-coagulation factor VIII antibodies) (30–41). F(ab)′_2_-mediated neutralization of inflammatory cytokines, chemokines, and complement fragments, along with regulation of immune cell apoptosis might contribute to the conversion of pro-inflammatory to anti-inflammatory conditions. Meanwhile, a possible explanation for the fact that only high-dose IVIg can exert anti-inflammatory effects is that a sufficient amount of antigen-specific IgG is required to achieve therapeutic activity.

However, as previous research revealed that preparations with purified Fc fragments of IgG had an undamped effect compared with intact IgG for some indications, F(ab)′_2_-mediated mechanisms alone may not be sufficient for the extensive immunomodulatory effects of high-dose IVIg therapy (42–44). Therefore, Fc-mediated mechanisms may play a decisive role in these processes.

### Fc-Mediated Mechanisms

**Figure 2:**
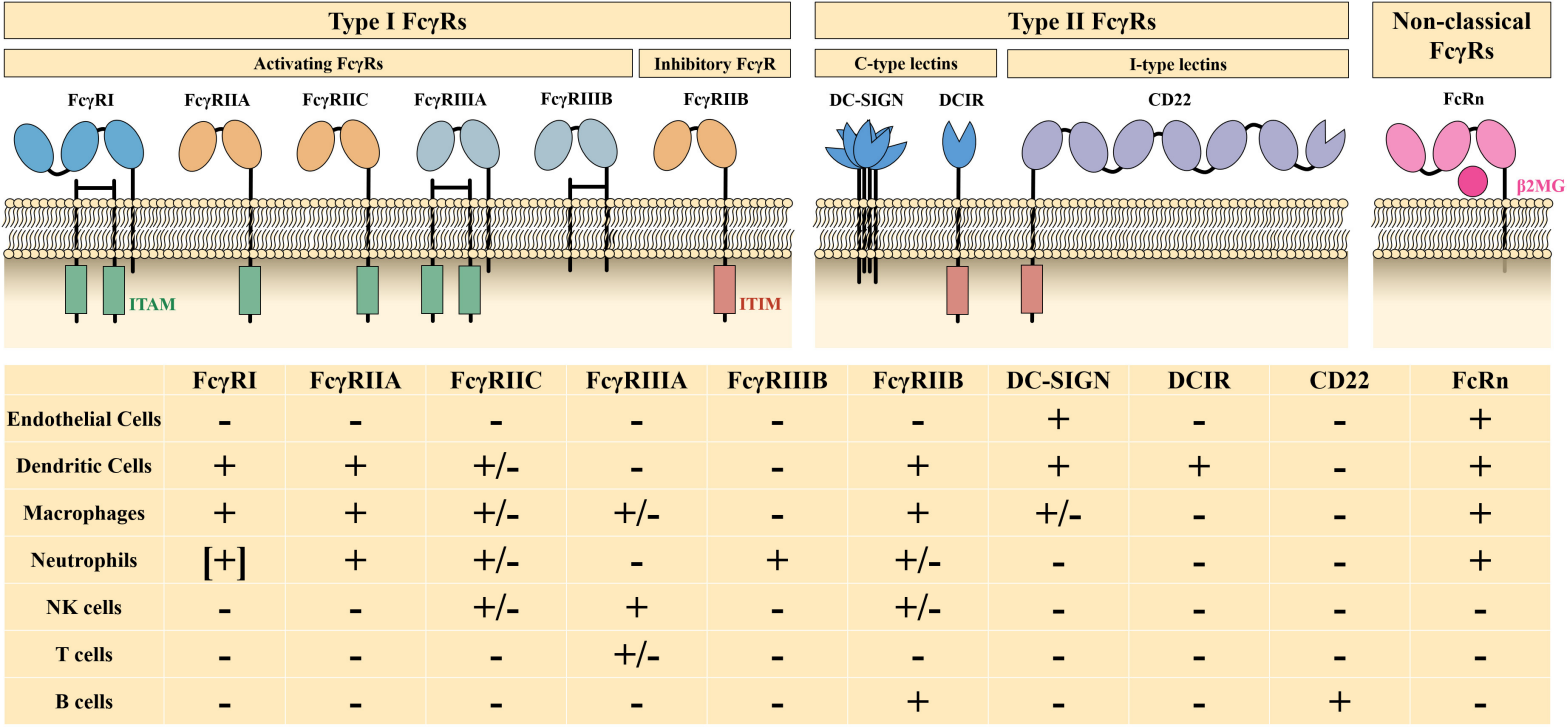
Characteristics and distribution profiles of human FcγRs. ITAM, immunoreceptor tyrosine-based activation motif; ITIM, immunoreceptor tyrosine-based inhibition motif; β2MG, β2-microglobulin; +, generally expressed on cells; –, not expressed on cells; [+], expressed on induced cells, +/–, expressed on specific genotype or subset of cells. Part of this figure has been adapted with permission from Springer Nature Customer Service Centre GmbH: Springer Nature: Nature Reviews Immunology, 13(3):176-89: Intravenous immunoglobulin therapy: how does IgG modulate the immune system?, by Inessa Schwab et al., 2013.

The Fc fragment of IgG is responsible for binding to Fc receptors (FcRs) and complement (e.g., C1q, C3b, and C4b). IgG-specific Fc receptors (FcγRs) belong to type I FcRs and are classified into activating FcγRs (FcγRI, FcγRIIA, FcγRIIC, FcγRIIIA, and FcγRIIIB) and an inhibitory FcγR (FcγRIIB), depending on their intracellular motif and the function they mediate. Type II FcRs, including C-type lectins (DC-SIGN, DCIR), type I lectins (CD22), and non-classical FcRs, such as FcRn and FCRL, also act as effector targets of IVIg (Figure 2). The Fc-mediated mechanisms of high-dose IVIg therapy include saturation of activating FcγRs and FcRn, upregulation of the inhibitory FcγRIIB, and scavenging of complement molecules.

#### Saturation and Limiting Access of Immune Complexes to Activating FcγRs

Given the central role of activating FcγRs in mediating excessive antibody-dependent effector functions, a possible explanation of the potential anti-inflammatory function of high-dose IVIg lies in that it significantly raises the concentration of IgG above the normal plasma levels and contributes to a functional blockade of FcγRs, limiting access of immune complexes to these activating receptors. Moreover, IgG from IVIg preparations has been proven to be active through inhibitory ITAMi signaling in immune regulation (45).

The role of dimeric IgG in IVIg preparations is emphasized by an enhanced understanding of the optimal IgG form to interact with activating FcγRs. In physiological conditions, monomeric IgG1 is more likely to bind FcγRI with high affinity rather than to other FcγRs with low affinity (46). Although monomeric IgG at therapeutic levels are able to saturate low-affinity FcγR in a dose-dependent manner, it has been suggested that the small amounts of dimeric IgG bind with higher avidity to FcγRs, and therefore constitute the main active component of IVIg mediating FcγR blockade, explaining the immunomodulatory function of high-dose IVIg in ITP, GBS, and CIDP patients (47–51).

#### Saturation of FcRn and Enhanced Clearance of Pathogenic Antibodies

Endogenous pathogenic autoantibodies, mainly IgG, play a predominant role in the immunopathogenesis of many autoimmune and infectious diseases. A hallmark characteristic of long serum half-life of endogenous pathogenic IgG is mainly due to FcRn. FcRn is a non-classical FcR that can interact with IgG in a pH-dependent manner, which is sufficient in rescuing IgG from lysosomal degradation and recycling it to the cell surface (52, 53). High-dose IVIg therapy leads to an increased clearance rate of monoclonal antibodies and pathogenic IgG in different mouse models, whereas this effect is not observed in FcRn deficient models, indicating that FcRn is involved in therapeutic effects of IVIg therapy (54, 55). Influences of FcRn gene polymorphisms on different IgG kinetics and therapeutic effects of IVIg among patients have been observed (56–58). Pharmacokinetic models predicted that the therapeutic effects of IVIg relate to total serum IgG concentration and FcRn-binding capacity (59). The substantial increase in IgG concentration may saturate FcRn and reduce the half-life of pathogenic antibodies, contributing to the anti-inflammatory mechanism of high-dose IVIg.

#### Upregulation of the Inhibitory FcγRIIB and Increase in the Activation Threshold

A balance between activating and inhibitory FcγRs is critical for a well-regulated immune response, and a disbalance markedly influences immunopathology in autoimmune and infectious diseases. FcγRIIB is the only ITIM-containing FcγR and negatively regulates many aspects of immune and inflammatory responses. After efficient high-dose IVIg therapy, an upregulation of FcγRIIB on immune cells is considered to contribute to the anti-inflammatory process (43, 60, 61). However, evidence of underlying molecular mechanisms remains sparse and this mechanism of therapeutic action is therefore controversial.

In the last decade, a prevailing theory from murine studies argued that a minor portion of IgG, with specific sialylation of the Asn297-linked glycan structure on the Fc fragment, is essential for anti-inflammatory activities of IVIg *via* direct interaction with myeloid regulatory cells expressing SIGN-R1 (mice) or DC-SIGN (human) (44, 62, 63). Subsequent research revealed that administration of sialylated IgG resulted in the production of IL-33 from hDC-SIGN^+^ macrophages or dendritic cells and ensuing expansion of IL-4-producing basophils, and these cytokines led to FcγRIIB expression on effector cells (64, 65). However, other studies have challenged this hypothesis. The evidence of a direct interaction between sialylated IgG and DC-SIGN is not fully supported in the literature (66, 67). Moreover, there is conflicting evidence suggesting that the sialylated Fc fragment of IgG is dispensable for the anti-inflammatory mechanisms of high-dose IVIg (68–72). It should be noted that all evidence of this hypothesis derives from murine studies, which might not be readily translated to human conditions. These controversial results warrant further research to address the mechanisms and molecular basis of high-dose IVIg upregulation of the inhibitory FcγRIIB.

#### Scavenging of Complement Fragments and Inhibition of Complement System Activation

The complement system is activated through three pathways, namely the classical pathway, the lectin pathway, and the alternative pathway. Besides the F(ab)′_2_-mediated neutralization of complement C3a and C5a, the interaction between the Fc fragment and complement C1q, C3b, and C4b in a dose-dependent manner, contributes to immunomodulatory effects of IVIg on the classical complement pathway (73–75). The binding site of C3b/C4b is located on the residues 381–390 of the CH3 domain of the IgG Fc fragment, while the residues 318–322 of the CH2 domain are responsible for the binding of C1q (76, 77). The binding domains may react with different N-glycan sialylation patterns on the IgG structure and result in distinct anti-inflammatory effects through the complement pathway (78).

As an extremely complex preparation, IVIg contains a large number of bioactive moieties, and the entirety of effects from IVIg is therefore not fully understood yet. The proposed antigen-specific F(ab)′_2_-mediated mechanisms and unspecific Fc-mediated mechanisms are not mutually exclusive, but is more likely to regulate the immune system in synergy, giving rise to the immunomodulatory effects of high-dose IVIg in specific clinical settings.

## The Immunopathological Hypothesis of Viral Pneumonia

Although an active immune response is essential for pathogen elimination in acute respiratory viral infections, excessive defensive reactions might wreak havoc on healthy cells and tissues. Complications or mortality of respiratory viral infections are often associated with excessive production of pro-inflammatory cytokines and ensuing multiple organ dysfunction. Although the immunopathogenesis of SARS-CoV-2 has not yet been fully described, the histopathological evidence strongly suggests a critical role of an excessive immune response in mediating extensive damage of the lung and other organs, similar to previous observations in SARS, MERS, influenza, and RSV disease, where hyper-inflammatory responses have been shown to be involved in the lung pathology. In this section, we review how a dysfunctional immune response may cause immunopathology in severe viral pneumonia, resulting in the current understanding of IVIg therapy in modulating the hyper-inflammatory conditions.

### Cytokine Storm and the Role of IVIg

The cytokine storm syndrome is a form of systemic inflammatory response common to severe acute viral pneumonias, and its presence has also been suggested in severe cases of COVID-19. There is a correlation between severity of the cytokine storm and prognosis of severe illnesses (13). At the initiation of infection, the host cells detect viruses through pattern recognition receptors (PRRs), which in turn triggers an interferon (IFN) response and produces other pro-inflammatory mediators such as cytokines and chemokines, informing both innate and adaptive immune system to respond appropriately to infectious pathogens. A physiological cytokine and chemokine response induced by viruses is a sprawling network, which involves endothelial cells, mononuclear macrophages, dendritic cells, natural killer cells, and lymphocytes, contributing to pathogen clearance and immune protection. However, an uncontrolled positive feedback involving all the relevant players leads to pathogenic hyper-inflammation and can cause extensive damage to tissues.

#### Endothelial Cells

Endothelial cells can secrete inflammatory mediators, including cytokines, chemokines, histamine, matrix metalloproteinases, and adhesion molecules, which can lead to inflammatory cell infiltration and tissue damage, and plays an essential role in inflammation, hemostasis, and angiogenesis.

SARS-CoV-2 infects host cells using the ACE2 receptor, which is widely expressed on endothelial cells. Endothelial cell infection and endotheliosis have been observed in some COVID-19 patients (79). Direct viral infection of the endothelium may result in extensive endothelial dysfunction and disseminated intravascular coagulation (17, 79). In addition, abnormal levels of complement factors and the deposition of terminal complement complex also suggested that the activation of complement pathways may contribute to endothelial damage and the associated pro-coagulant state in COVID-19 (80, 81).

Although vasculitis and autoantibodies against endothelial cells have been reported and detected in SARS patients, direct endothelial cell infection hasn't been demonstrated yet (82–84). These differences in endothelial infections may contribute to the different immunopathology between SARS-CoV-1 and SARS-CoV-2. In influenza, the central role of the pulmonary endothelium for the regulation of innate immune cell recruitment into the lungs, along with the production of excessive pro-inflammatory cytokines and chemokines, has been demonstrated (85–87).

The anti-inflammatory effect of high-dose IVIg on endothelial cells is mainly inferred from *in vitro* observations. High-dose IgG has been shown to specifically and completely inhibit TNF-α-induced secretion of pro-inflammatory cytokines (e.g., IL-6, G-CSF, and IL-1β) and production of E-selectin in cultured human coronary artery endothelial cells (88, 89). These effects may predominantly be mediated by F(ab)′_2_-mediated mechanisms (88, 89). The expression of endothelial adhesion molecules is also reduced by co-culturing endothelial cells with an IVIg preparation (90). Additionally, the inhibitory effects of the classical complement pathway by high-dose IVIg may offer a potential protective effect on virus-induced endothelial damage.

#### Dendritic Cells

Dendritic cells (DCs) are antigen-presenting cells of the innate immune system. Plasmacytoid dendritic cells (pDCs) are unique sentinel cells that can recognize a virus through PRRs, and enhance the secretion of IFN (91, 92).

Data from COVID-19 patients and a ferret model reveal low levels of IFN with contrastingly high levels of IL-6 and a strong chemokine response, suggesting a similar hyper-inflammatory response pattern to SARS and MERS (93). Several SARS-CoV and MERS-CoV proteins have been shown to antagonize the IFN response in order to reach high viral titers after early infection (94–98). Delayed and massive IFN signaling is suggested to further orchestrate extensive inflammatory monocyte-macrophage responses and induce T cell apoptosis, resulting in a dysfunctional immune response and cytokine storm during SARS-CoV and MERS-CoV infection (99–101).

The immunomodulatory effects of IVIg on DCs differ depending on dosage. IVIg accelerates maturation at physiological doses, but inhibits the maturation, activation, and function at therapeutic doses (102). High-doses IVIg treated immature DCs secrete increased levels of anti-inflammatory cytokines, and decreased levels of pro-inflammatory cytokines (103). C-type lectins expressed on DCs are considered as possible effector targets of IVIg preparations, however, the exact mechanism of action of IVIg on DCs remains controversial. As stated, interactions between Fc-sialylated IgG and DC-SIGN failed to reproduce in human cells, and the expression of FcγRIIB remains stable on DCs after IVIg treatment (104). Another C-type lectin, DRIC, has also been identified to bind to Fc-sialylated IgG and further mediates the induction of Treg cells in a mouse model (105). Additionally, an increased accumulation of lipids and decreased expression of MHC-II, CD40, CD80/CD86, and IFN-γR2 on DCs upon high-doses IVIg treatment might suppress antigen presentation and allogeneic T-cell stimulatory capacity, contributing to a negative regulation of the immune response (104, 106, 107).

#### Monocyte-Macrophages

Whether infiltrating inflammatory monocyte-macrophages (IMM) are beneficial or deleterious after a viral infection is mainly dependent on their ability to secrete inflammatory cytokines and chemokines. A dysregulated cytokine response can also promote excessive activation of IMM, leading to immunopathological effects on healthy tissues.

Macrophage activation syndrome (MAS) was reported in several COVID-19 patients with severe respiratory failure, and the production of associated pro-inflammatory cytokines (IL-6 and TNFα) might contribute to hyper-inflammatory conditions (108). It was also detected that SARS-CoV-2 infects macrophages and triggers secretion of IL-6, which might contribute to lymphocyte apoptosis, suggesting an excessive infiltration and intense pro-inflammatory activity of IMM in COVID-19 patients (109).

Increased pathogenic IMM influx has been consistently observed in severe or lethal SARS patients, along with mice and Chinese rhesus macaque models (101, 110). The activation of macrophages by SARS-CoV occurs by TLR2 ligand recognition, subsequent activation of the NF-κB pathway and secretion of cytokines, contributing to the immunopathology of SARS (111–113).

High-dose IVIg therapy is widely used for treatment of patients with autoimmune diseases complicated by MAS (114–118). For inhibitory effects of IVIgs, functionally activating FcγRs are required to acquire a cross-tolerant state of mouse macrophages (119). *In vitro*, IVIg inhibits the production of pro-inflammatory cytokines by M1 macrophages and triggers macrophage polarization via FcγRIII-mediated mechanisms (120). IVIg limits the differentiation of macrophages through inhibition of GM-CSF-driven STAT5 activation by FcγR-dependent mechanisms (119). An increased production of anti-inflammatory cytokines of LPS-stimulated monocytes (especially IL-10) is further enhanced by IVIg through phosphorylation of ERK1/ERK2 and P38/MAPK *via* FcγRIIA-mediated mechanisms (121).

#### Neutrophils/Granulocytes

Neutrophils are the most abundant type of granulocytes in peripheral blood and constitute an essential part of innate immunity. The release of cytokines, nitric oxide, reactive oxygen species (ROS), and neutrophil extracellular traps (NET) by neutrophils can help to neutralize pathogens. Paradoxically, however, neutrophil reactivity can also enhance tissue damage in hyper-inflammatory conditions such as severe virus infection.

Excessive neutrophils and NET production are considered a potential predictor of prognosis in influenza (122, 123). Similarly, it has been considered that neutrophils in SARS-CoV-2 may induce NETs and contribute to organ damage (124, 125). High levels of NET biomarkers (e.g., cell-free DNA, myeloperoxidase-DNA, and citrullinated histone H3) are detected in severe COVID-19 patients and may contribute to cytokine storm and respiratory failure (125).

Both Fc and F(ab)′_2_ fragments account for the anti-inflammatory activity on neutrophils. In patients with Kawasaki disease, IVIg therapy reduces the activation and nitric oxide production of neutrophils (126). *In vivo* studies revealed that IVIg inhibits neutrophil recruitment and activation through the activation of SHP-1 *via* FcγRIII-mediated mechanisms (127). Furthermore, IVIg contains anti-Siglec autoantibodies that can regulate neutrophil apoptosis in a dose-dependent cytotoxic manner *via* F(ab)′_2_-mediated mechanisms (33, 128, 129). Interestingly, high-dose sulfo-IVIg preparations significantly reduce NET formation *in vitro* as well as in rat models (130).

#### Natural Killer Cells

Natural killer (NK) cells are innate lymphocytes that play an essential role in the control of respiratory viral infections. Within days after a respiratory viral infection, NK cells are activated, recruited to the lung, and act as effector cells in a classical FcγR-mediated function, namely antibody-dependent cytotoxicity (ADCC). However, a dysfunction of NK cells may also be responsible for immunopathogenesis during infection.

Increased inhibitory receptor NKG2A expression on NK cells and reduced production of CD107a, IFN-γ, IL-2, and TNF-α, indicate a functional exhaustion of NK cells during SARS-CoV-2 infection, similar to that observed in SARS (131). In patients with severe SARS, the number of NK cells and levels of the functional NK-marker KIR2DL3 were significantly lower than in patients with mild SARS, mycoplasma pneumonia, or healthy controls, manifesting a correlation between the severity of SARS and the number and function of NK cells (132). During influenza virus and RSV infection, virus-induced apoptosis or modulation of NK cell cytotoxicity has also been proposed to serve as a viral evasion strategy, and eventually skew the inflammatory profile of the immune system (133, 134).

After high-dose IVIg therapy, a reduction in number and cytotoxic activity of NK cells is observed in patients with autoimmune diseases (135–137). The dose-dependent FcγRIII blockade and circulating NK cells decline occurred following IVIg treatment, suggesting that inhibition of NK cells by high-dose IVIg is associated with the saturation of activating FcγRs (135). The inhibitory effects of IVIg therapy on NK cells may, however, not impair its functions for controlling viral infections and malignancies (138).

#### CD4+ T Lymphocytes

Cytokines secreted by different subclasses of CD4+ T cells trigger the immune response to pathogens and maintain immune homeostasis. However, an imbalance of Th1, Th2, Th17, and Treg subclasses is associated with dysfunctional cytokine responses during severe viral infections.

The profiles of serum cytokines revealed an increased concentration of Th17 cells in COVID-19 patients (139, 140). In severe SARS patients, the activation of Th1-related cytokines and chemokines (e.g., IL-1, IL-6, IL-12) was involved in hyper-inflammatory conditions (141). Furthermore, in MERS patients, the significantly increased release of pro-inflammatory cytokines from Th1 and Th17 during the acute phase was considered to be at least partly responsible for the immunopathology (142). Early abundant secretion of Th1- and Th17-related cytokines (e.g., IL-6 and IL-17) has furthermore been associated with complicated infections and mortality in severe influenza patients (143, 144).

In addition to mediating expansion of Tregs, which profoundly impacts immune cascades, high-dose IVIg has also been shown to inhibit the activation and subsequent production of cytokines by Th1 and Th17 cells in several clinical studies and *in vitro* experiments (145–158). Moreover, F(ab)′_2_ fragments, rather than Fc fragments, have been shown to retain the function of intact antibodies in inhibiting Th1 and Th17 (158). It has been proposed that IVIg may neutralize sphingosine-1-phosphate (S1P) receptors on the CD4+ T cell and downregulate the S1P1-mTOR signaling axis, thereby inhibiting the differentiation and infiltration of Th1 and Th17 cells while favoring Treg cells (158). Interestingly, monomeric IgA (mIgA) isolated from a IVIg preparation has been revealed to interfere with the STAT3 *via* its F(ab)′_2_ fragments and inhibits the differentiation and expansion of Th17 cells (159). It is noted that the proposed mechanism of functional T cell-modulation by IVIg also includes DC-mediated effects; IVIg-primed DCs can steer non-Treg cell precursors toward a Treg differentiation, and C-type lectins expressed on DC cells may be critical in this process (105, 160–162). Novel Treg epitope peptides (Tregitopes) on IgG provide a further explanation of the regulatory effects of IVIg on Treg cells *via* DC-mediated internalization of IVIg (163). Tregitopes are natural T cell epitopes of IgG, and the presentation of Tregitopes on DCs in the context of MHC-II can lead to the activation and expansion of Treg cells, reinforcing the immunoregulatory effects on other conventional T cells (164, 165).

#### CD8+ T Lymphocytes

CD8+ T cells contribute to pathogen clearance and immune protection. However, cytokines and cytotoxic granules produced by activated CD8+ T cells may exaggerate cytokine storms and are partially implicated in the immunopathology during respiratory viral infections (166, 167).

Both a substantial reduction of CD8+ T cell counts in peripheral blood, and hyperactivation with extensive expression of surface activation markers (HLA-DR and CD38) and cytotoxic granules in CD8+ T cells are commonly observed in COVID-19 patients (139, 168). An accompanying functional exhaustion of CD8+ T cells is furthermore detected in severe COVID-19 patients (131). Unlike influenza or RSV, the impaired function of CD8+ T cells during SARS-CoV-2 infection suggests that these cells may not be major contributors to the cytokine storm in severe COVID-19 patients (166, 167, 169). However, overactivation and subsequent functional exhaustion of CD8+ T cells may correlate with disease progression (131).

After effective high-dose IVIg therapy, the expression of HLA-DR and the proportion of extensive highly activated Vβ elements of CD8+ T cells decrease in patients with autoimmune diseases (170, 171). *In vitro* and animal model studies report similar inhibitory effects (172, 173). As no FcγRs are expressed on T cells, the modulation of IVIg on CD8+ T cells may largely depend on specific antibodies present in the IVIg preparation, which directly bind to T cells, or through interactions between APC and TCR signaling (174). For example, it has been shown that the anti-B07.75–84 peptide antibodies in IVIg may be able to inhibit HLA class I–restricted cytotoxicity of CD8+ T cells *via* their F(ab)′_2_ fragment (175). Apart from that, a saturation of activating FcγRs on APC results in reduced immune complex internalization and presentation, further contributing to the inhibitory effects of high-dose IVIg on CD8+ T cells (172, 173).

#### B Lymphocytes

With assistance from helper T cells and stimulation from cytokines, B cells are activated and differentiated into plasma cells to produce antibodies and contribute to humoral immunity (169, 176). Apart from the generation of antibodies, IL-6 produced by activated B cells is also considered as a possible booster of the cytokine storm and immunopathology in autoimmune disease (177, 178).

The rapid reduction of peripheral B cells is also a significant characteristic of severe COIVD-19 patients, yet the exact mechanisms underlying this accelerated lymphocyte loss remain unclear (17, 179). Although there is so far no reliable evidence correlating an overactivation of B cells with the development of cytokine storms during respiratory viral infections, the production of pro-inflammatory cytokines by B cells has been proposed to play a key role in the cytokine cascade in COVID-19 (180–182).

Effects of IVIg on B cells vary with the dosage: at low doses, IVIg induces proliferation of B cells and the production of antibodies (183, 184). On the contrary, high-dose IVIg inhibits the activation of B cells *via* both F(ab)′_2_-mediated mechanisms (e.g., neutralizing APRIL, BAFF, and Fas) and Fc-mediated mechanisms acting on inhibitory FcRs (e.g., FcγRIIB and CD22), as well as the TLR-9 signaling cascade.

FcγRIIB is the only classical FcγR expressed on B cells, and its elevated expression levels after effective high-dose IVIg therapy may be involved in inhibitory effects of IVIg on B cells (61, 185, 186). However, IVIg-induced upregulation of FcγRIIB in B cells is independent of classical FcγRIIB signaling intermediates, suggesting that it is likely a consequence rather than a cause of high-dose IVIg therapy (187, 188). It has been proposed that sialylated IgG binds to DC-SIGN in addition to CD23, a C-type lectin which is also a known low-affinity IgE receptor characteristically expressed on B cells, inducing the suppression of B cells (189). However, there has been disagreement as to whether there is a direct interaction between IgG and DC-SIGN/CD23 (67, 190). A further proposed mechanism of action of high-dose IVIg is the interaction between the sialylated Fc fragment of IgG and CD22, an I-type lectin expressed on B cells, which results in the activation of the ITIM signaling cascade, subsequently promoting B cell apoptosis (183, 191, 192). Except for FcRs, the TLR-9 signaling cascade may also be involved in inhibitory effects. IVIg could mimic the effects of MyD88 inhibitors and result in the suppression of the TLR-induced NF-κB signaling pathway and downstream production of cytokines (193, 194).

A “paradoxical” phenomenon has been observed, where large amounts of immature plasma cells were mobilized after high-dose IVIg therapy in patients with autoimmune diseases, suggesting *de novo* B cell activation. This is consistent with the immunomodulatory functions of IVIg at low doses (184, 195, 196). Although the potential roles of plasma blasts under different disease conditions remain unclear, this phenomenon highlights binary immunomodulatory effects of high-dose IVIg to humoral immunity.

To summarize, the cytokine storm observed during severe viral infection is associated with various immune dysfunctions. Several therapeutic interventions targeting the host immune response have been attempted. Although the proposed mechanisms of high-dose IVIg on the modulation immune cells and cytokine cascades are mainly derived from research into autoimmune diseases, its use in reversing a cytokine storm, in addition to providing passive immunity during severe viral infection, is supported by recent evidence.

### Antibody-Dependent Enhancement Phenomenon and the Role of IVIg

The humoral immune response to invading pathogens by producing neutralizing antibodies is crucial in the host adaptive immune system. Paradoxically, antibodies may provide an attractive means for a variety of viruses to enhance viral entry and replication in some cell types under certain conditions. The unique phenomenon that preexisting poor neutralizing antibodies facilitate viral access to FcγRs and lead to enhanced infection or immunopathology is also referred to as antibody-dependent enhancement (ADE). ADE has been implicated widely in flavivirus infections, such as WNV and DENV, and high-dose IVIg is commonly used in treating WNV encephalitis with satisfactory effect. However, little is known about ADE in respiratory virus infection (197–199).

#### ADE in SARS, MERS, Influenza, and RSV Disease

ADE is currently being considered as a potential contributor to the immunopathology of SARS. People who succumbed to SARS had significantly higher S glycoprotein-specific neutralizing antibodies in serum during the early stage of infection, indicating the possible presence of ADE (200). Similarly, recent results in SARS-CoV-infected Chinese rhesus macaques consistently showed that anti-spike IgG significantly amplified pro-inflammatory cytokine production in activated macrophages and enhanced pulmonary pathology. These effects could be reduced by the FcγRII blocking antibody (110). Similarly, it has been shown that infection of macrophages is enhanced by anti-SARS-CoV spike immune serum but eliminated by the FcγRII antibody, suggesting an FcγRII-dependent ADE of SARS-CoV (201). Molecular signaling analyses investigating FcγRII-mediated infection by SARS-CoV revealed that an intact cytosolic domain of FcγR was required, and FcγRIIA was more prone to ADE than FcγRIIB (202).

Although *in vivo* evidence of ADE in MERS is lacking, it has been shown that MERS-CoV infections depended on monoclonal antibody (MAb) concentration, binding affinity of MAb for the viral receptor DPP4, and tissue expressions of DPP4 and FcγR, providing a molecular basis of ADE in MERS-CoV (203). Likewise, the presence of sub-neutralizing titers of antibodies is also a contributing factor to the unfavorable outcome in influenza infection. Prophylactic treatment with monoclonal antibodies at a low dose following the H3N2 virus challenge in mice has shown enhanced cellular infiltration and lung pathology compared to the control group (204). In contrast, a high dose prophylaxis showed protective effects (204). Vaccinating pigs against H1N2 resulted in the generation of cross-reactive anti-pH1N1 HA2 antibodies, which exhibited poor neutralizing ability to the HA1 domain and enhanced pH1N1 infection and lung pathology (205). Besides influenza and potentially MERS, the ADE phenomenon has also been observed in RSV infection (206). It has been shown that RSV infections are significantly enhanced in NK cells previously incubated with sub-neutralizing titers of RSV-specific antibodies *in vitro*, promoting IFN-γ production of NK cells (207). It remains unclear whether such *in vitro* enhancement of RSV infection has a correlation with *in vivo* disease. Nevertheless, the Fc-mediated antibody effector functions are believed to participate in the antibody-dependent enhancement of RSV disease (206, 208).

#### ADE in COVID-19 and the Role of IVIg

To date, the exact role of ADE in SARS-CoV-2 infection remains unclear. Regarding concerns that reappearance of cross-reactive antibodies from other serotypes of coronavirus may enhance the current infection, the risk of ADE needs to be clarified when treating COVID-19 patients (203, 209).

As described above, the occurrence of ADE requires binding of the virion to antibodies at a sub-neutralizing concentration and subsequent uptake by FcγR-bearing cells (210). To inhibit potential phenomenon of ADE in COVID-19, high-dose IVIg therapy is therefore proposed (211). First, it is unlikely to have preexisting anti-SARS-CoV-2 antibodies in IVIg preparations early in the novel epidemic. Additionally, a high concentration of non-neutralizing antibodies, rather than a regular or diluted dose, would integrally saturate activating FcγRs and FcRn, which is beneficial to limit access of immune complexes and enhance clearance of inimical antibodies, to ultimately provide immunomodulatory effects in severe COVID-19 patients (211, 212).

## Lessons From Previous and Current Viral Pneumonias

Based on its efficacy and supporting mechanisms in modulating the inflammatory response and improving serum IgG levels, high-dose IVIg therapy is considered for treatment of several severe viral infections. Potential protective effects of IVIg preparations were previously shown in influenza- and RSV-infected animal models (213–218). In this section, we review the previous clinical application of high-dose IVIg therapy in treating viral pneumonia such as SARS, MERS, influenza and RSV disease, as well as its current application in COVID-19 (Table 2).

**Table 2:** Description of studies within IVIg treated severe acute viral pneumonia patients.

**Disease**	**Study design**	**Population, Study *N***	**Study observations/Clinical outcomes**	**Conclusions/Inferences**	**References**
	**IVIg dosage**	**Co-treatments**			
COVID-19	Retrospective 20 g/d, ? d	Adult, *N* = 58, 100% with IVIg Steroids ? %, antivirals: all^*^	- Survivors had received earlier IVIg therapy (2.3 vs. 3.4 days) - 28-day mortality was 23.3% vs. 57.1% (initiated IVIg within 48 h vs. after 48 h) of severe or critical ill COVID-19 patients	- Benefit for early initiation of IVIg	(219)
COVID-19	Retrospective N.D.	Adult, *N* = 53, 29 (55%) with IVIg Steroids 70%, antivirals 98%^†^	−30-day mortality was 93.1% vs. 79.2% (IVIg group vs. others, *P* = 0.51) of COVID-19 patients with ARDS	- No significant effect of IVIg on survival in ARDS patients	(220)
COVID-19	Retrospective 20 g/d, ? d	Adult, *N* = 10, 100% with IVIg Steroids: all, antivirals: all^‡^	−7 patients improved and 4 of them discharged after treatment - Temperature, SPO2 (from 90.5 to 97.5%), PaO_2_/FiO_2_ (from 129.3 to 340.8 mmHg), lymphocyte counts (from 0.59 to 1.36 × 10^9^/L), CRP level (from 49.94 to 14.58 ng/L), APACHE II scores (from 9.1 to 5.5), and pulmonary lesion were significant improved	- Benefit for higher dose of IVIg and corticosteroid combined therapy	(221)
COVID-19	Case report 25 g/d, 5 d	Adult, *N* = 3, 100% with IVIg Steroids 33.3%, antivirals 66.7%^§^	- Clinically improved shortly after therapy with temperature normalized within 1–2 days and dyspnea relieved within 3–5 days - The chest radiographic scans and laboratory measures also displayed normalization after treatment	- Benefit for early initiation of high-dose IVIg	(16)
SARS	RCT 5 g/d, 5 d^¶^	Adult, *N* = 44, 23 (52%) with IVIg Steroids: all, antivirals: none	- The serum IgG increased slightly from 27.3 to 28.2 g/L in IVIg group and decreased significantly from 28.4 to 23.3 g/L in control - No significant difference in the fatality rates (18.1% vs. 23.8%) and the nosocomial infection rates (47.8% vs. 52.4%) between IVIg and control	- IVIg effectively maintain the IgG concentration - No significant effect of IVIg on the fatality rate and nosocomial infection rate	(222)
SARS	Retrospective 1 g/kg/d, 2 d	Adult, *N* = 76, 40 (53%) with IVIg Steroids 90.8%, antivirals 93.4%^‖^	- The leukocyte counts, and platelet counts significantly increased after treatment (from 2.6 to 4.3 × 10^9^/L and from 104 to 141 × 10^9^/L) among 22 patients who received IVIg therapy without steroids	- Benefit of IVIg in controlling leukopenia and thrombocytopenia	(223)
SARS	Retrospective 0.2–0.4 g/kg/d, 3 d	Children, *N* = 10, 100% with IVIg Steroids: none, antivirals: none	- The WBC counts significantly increased (from 2.72 to 5.81 × 10^9^/L), and the temperature normalized in 1, 6, 9, 10 of 10 patients on days 1, 2, 3, 4 after initiation of IVIg therapy - The chest radiographic imagines significantly rapid absorption on days 10 vs. days 13.5 of historically controlled pediatric patients	- Benefit of IVIg in controlling leukopenia and improving lung lesions absorption	(224)
SARS	Retrospective 5 mL/kg/d, 3 d	Adult, *N* = 12, 100% with Pentaglobin Steroids: all, antivirals: all^‖^	- The radiographic scores were significantly reduced from 9.5 to 7.5, 6, and 6 on days 1, 5, 6, and 7 - Oxygen requirement reduced from 2.5 to 1 and 0.5 L/min on days 1, 6, and 7, respectively	- Benefit of IgM-rich IVIg therapy in steroid-resistant SARS patients	(225)
MERS	Case report 0.1 g/kg/d, 2 d	Adult, *N* = 1, 100% with IVIg Steroids: no, antivirals: no	- The effect of IVIg therapy cannot be evaluated	- Inconclusive	(226)
Influenza	RCT 0.4 g/kg, once	Adult, *N* = 34, 17 (50%) with IVIg Steroids: none, antivirals: all^**^	- Greater rate of viral load reduction in Flu-IVIg group (from 5.83 to 3.30) than IVIg group (from 5.34 to 4.67), - No significant difference of the cytokine (IFN-α2, IL-1α, IL-6, IL-10, IL-15, IL-1ra, MCP-1, MIP-1α, GM-CSF, TNF-α) on day 5 - Flu-IVIg was the only factor that independently reduced mortality (OR 0.14; 95% CI 0.02-0.92, *P* = 0.04)	- Benefit of IVIg in controlling cytokine storm - No significant effect of IVIg on viral clearance and mortality	(227)
Influenza	RCT 1 g/kg/d, 2d	Children, *N* = 50, 25 (50%) with IVIg Steroids: all, antivirals: all ^††^	- Temperature normalization (80% vs. 50%) and reduction of cough (68% vs. 44%), rhinorrhea (52% vs. 32%), tachypnea (80% vs. 32%), respiratory sounds [wheeze (64% vs. 28%) and rhonchi (76% vs. 32%)] and chest radiographic lesions (76% vs. 28%) in IVIg group compared with control group after initiation of 5 days	- Benefit of IVIg therapy in the prognosis of pediatric patients with severe H1N1	(228)
Influenza	RCT 1 g/kg/d, 2d	Children, *N* = 24, 12 (50%) with IVIg Steroids: all, antivirals: all ^††^	- Temperature normalization (83% vs. 50%) and reduction of dyspnea (75% vs. 42%), respiratory sounds (67% vs. 50%), chest radiographic lesions (75% vs. 42%) and cardiac enzymes normalization (50% vs. 33%) in IVIg group compared with control group after initiation of 5 days	- Benefit of IVIg therapy in the prognosis of pediatric patients with severe H1N1	(229)
RSV	Retrospective 0.2–0.4 g/kg, once	Immunocompromised, *N* = 56 14 (25%) with IVIg Steroids: none, antivirals 64.3%^‖^	- Hypogammaglobulinemia was significantly associated with fatal outcome (OR 11.76; 95% CI 1.38–11.26, *p* = 0.007) while treatment with IVIg was not able to reverse this association - Only ribavirin therapy (OR 0.14; 95% CI 0.02–0.92, *p* = 0.02) represented a protective factor	- No significant effect of IVIg on fatal outcome and hypogammaglobulinemia	(230)
RSV	Retrospective 2 g/kg/d, 2–4 d	Immunocompromised, *N* = 49 22 (84.6%) with IVIg Steroids: 32.7%, antivirals 92.3%^‖^	- Progression from URTI to LRTI occurred in 15%, and only 1 patient died from RSV disease - IVIg for LRTI was generally well-tolerated	- Benefit of IVIg therapy in preventing development of URTI and prognosis of LRTI	(231)
RSV	Prospective 0.5 g/kg, every other day	Immunocompromised, *N* = 14 100% with IVIg Steroids: none, antivirals: all^‖^	−10 of 14 patients were resolved with URTI, while the other 4 (28.6%) developed LRTI, and 2 (14.3%) patients died. Compared to other research results, with 32% of patients developing LRTI and 88% of whom died in only aerosolized RBV treatment	- Benefit of IVIg therapy in preventing development of URTI and prognosis of LRTI	(232)

*ARDS, acute respiratory distress syndrome; N.D., no data; SPO_2_, surplus pulse O_2_; CRP, C-reaction protein; APACHE II, acute physiology and chronic health evaluation score; URTI, upper respiratory tract infection; LRTI, lower respiratory tract infection*.

* *All patients were treated with Arbidol*.

† *95% patients were treated with Ribavirin, 26% with Oseltamivir, and 16% with Arbidol*.

‡ *80% patients were treated with Lopinavir/ritonavir, 60% with interferon, and 30% with Arbidol*.

§ *Patients 1 were treated with Oseltamivir, and Patients 3 with steroids and Lopinavir/ritonavir*.

¶ *Patients received IVIg at 2.5–10 g/d for 2-13 d, average to 5 g/d for 5 d*.

‖ *These patients were treated with Ribavirin*.

** *All patients were treated with Oseltamivir, and 8.8% with zanamivir*.

†† *All patients were treated with Oseltamivir*.

### IVIg in Treating SARS

During the global outbreak of SARS in 2003 caused by SARS-CoV, different therapeutic modalities (e.g., α-interferon, ribavirin, LPV/r, corticosteroids, convalescent plasma, and IVIg) were empirically used (233). Although strong evidence recommending the administration of IVIg therapy is lacking and a previous systemic review concluded that the evidence for efficacy of improving prognosis with IVIg therapy remained inconclusive, some studies commented that patients seemed to improve upon IVIg treatment (233, 234).

In a randomized controlled trial, IVIg therapy efficiently improved the serum IgG concentration of severe SARS patients compared to the control group (222). However, possibly due to the inadequately controlled dosage of glucocorticoids and IVIg therapy, which were given at the discretion of clinicians, there were no significant differences in the fatality rates and the rates of nosocomial infection of severe SARS patients between the IVIg group and the control group (222).

Apart from hypogammaglobulinemia, two further common features of severe SARS are leukopenia and thrombocytopenia, and these features seemed to improve in SARS patients upon IVIg therapy (223, 234). The peripheral WBC and platelet count of severe SARS patients significantly increased after receiving IVIg therapy without steroids, suggesting a potential role of IVIg in controlling leukopenia and thrombocytopenia in SARS patients (224). Furthermore, significant clinical improvement in pediatric SARS patients was noted after IVIg therapy (224). A declining WBC significantly recovered after IVIg therapy, and the temperature of most patients normalized within 3 days. Compared to the historical controlled group, a significantly shorter time of chest radiographic absorption in these patients was observed and indicated the potential efficacy of IVIg in clinical improvement and absorption of lung lesions.

Interestingly, an IgM-enriched IVIg preparation also showed significant beneficial effects in deteriorating SARS patients who failed corticosteroid and ribavirin treatment (225). A significant improvement regarding the oxygen requirements and radiographic scores were observed after pentaglobin therapy, and most patients recovered. It has been proposed that abnormal cytokine levels (e.g., IL-6 and TNF-α) are correlated with poor prognosis in SARS patients, and that the inhibitory effects of pentaglobin on cytokine release might represent an essential mechanism of action in the treatment of SARS (225, 235).

### IVIg in Treating MERS

MERS is a highly fatal respiratory disease caused by MERS-CoV, with two major historic outbreaks in 2012 and 2015. The rapid deterioration of health in a large number of patients made it unrealistic and unethical to perform randomized controlled treatment trials. Therefore, the current availability of strong evidence is minimal, and only a few case series reported administration of IVIg late in the course of MERS. These studies discussed the possible efficacy of IVIg in reversing severe thrombocytopenia through immunomodulatory mechanisms (226, 236).

### IVIg in Treating Influenza

Although influenza has been around for centuries, severe influenza remains a health challenge for humans. The complications or deaths are usually associated with the extensive induction of pro-inflammatory cytokines in severely affected influenza patients (166). Immunomodulatory strategies or treatments, including amongst others corticosteroids, PPARs agonists, S1P1 receptor 1 agonists, antioxidants, and IVIg therapy, have been widely considered as adjunctive treatments for cytokine storms in influenza (237).

In a previous randomized controlled trial, patients infected with the severe 2009 pandemic influenza A (H1N1) were randomized to receive 0.4 g/kg for one dose of Flu-IVIg (anti-influenza hyperimmune intravenous immunoglobulin with high HAI antibodies level) or IVIg therapy (227). Patients who received Flu-IVIg showed a greater rate of viral load reduction than the IVIg group. However, there was no significant difference in the cytokine profiles (e.g., IFN-α2, IL-1α, IL-6, IL-10, IL-15, IL-1ra, MCP-1, MIP-1α, GM-CSF, TNF-α) between the IVIg and Flu-IVIg group on day 5. Subgroup multivariate analysis of patients receiving treatment within 5 days of symptom onset revealed that Flu-IVIg therapy, rather than IVIg therapy, was the only factor that independently reduced mortality.

In contrast, other RCTs using IVIg treatment at high doses have shown improved survival. In two similar studies, pediatric patients with the severe 2009 pandemic influenza A (H1N1) were randomized to receive standard care or standard care plus 1 g/kg/d high-dose IVIg for 2 days (228, 229). Significant clinical improvement with regards to temperature normalization, reduction of cough, rhinorrhea, tachypnea, respiratory sounds (namely wheeze or rhonchi), and chest radiographic lesions were observed in the IVIg group compared to the control group, revealing a possible clinical benefit of high-dose IVIg therapy for the improvement of clinical symptoms in pediatric patients with severe influenza A H1N1 (228, 229).

### IVIg in Treating RSV Infection

RSV is a major respiratory pathogen that causes extensive respiratory symptoms, including both upper and severe lower respiratory tract infection (URTI/LRTI) in infants, the elderly, and immunocompromised individuals (238). Available therapies are limited to RBV, IVIg, and palivizumab, but the use of IVIg for treatment of RSV infections remains controversial (239).

A study showed that hypogammaglobulinemia was a significant risk factor for fatal outcomes of RSV infections in a hematology and transplant unit, and treatment with IVIg at regular doses was unable to reverse the poor prognosis (230). By contrast, oral RBV in combination with high-dose IVIg therapy (rather than lower doses), have been potentially attributed to the improvement of survival in RSV-infected allogeneic hematopoietic stem cell transplanted (HSCT) patients (231). The dosage of IVIg might be the critical factor for efficacy of therapy, since the combination of high-dose IVIg and aerosolized RBV therapy has also been shown to be a safe and promising approach to prevent progression of RSV infections immunocompromised patients (232).

Several published studies suggest that a combined therapy of RBV and IVIg improves the outcome of HSCT and leukemia patients with RSV-URTI, and additional recommendations are given for high-risk patients with RSV-LRTI (240–242). However, it is noted that most studies on IVIg efficacy are limited to pediatric or HSCT patients, which may not explicitly be recommended in all situations (243).

In summary, IVIg therapy exhibits different levels of potential clinical benefits in SARS, MERS, influenza, and RSV infections, though currently there is no high-level evidence to support IVIg use in these infections. Of note, the presented results are partly limited to pediatric and immunocompromised patients, and the clinical benefits of IVIg therapy may vary in different patient populations (e.g., infants, the elderly, and immunocompromised individuals). It is also noteworthy that most studies for these viral infections show possible benefits from IVIg therapy at higher doses, which indicates that a high dosage might be key for clinical efficacy. Considering the immunomodulatory effects of high-dose IVIg on the excessive immune cascade, high-dose IVIg therapy, as stated above, may be considered a potential adjunctive treatment for severe COVID-19 patients.

### IVIg in Treating COVID-19

The national diagnosis and treatment protocol for COVID-19 (Trial Version 7) and recommendations from the Peking Union Medical College Hospital have suggested the application of IVIg therapy in severe and critically ill COVID-19 patients (4, 18). Several observational and interventional studies were conducted to evaluate the efficacy of IVIg.

A retrospective study (*n* = 58) in China included severe and critical ill COVID-19 patients that were administered IVIg therapy after admission (219). IVIg therapy was given at 20 g daily, and 23 of 58 (39.6%) patients died within 28 days. For retrospective analysis, patients were split into two groups, based on whether they received IVIg administration within or after 48 h of admission to the intensive care unit (ICU). The results showed that application of IVIg within 48 h of admission to ICU reduced the use of mechanical ventilation, shortened duration of ICU and hospital stay, and improved 28-day survival (219). The 28-day mortality of the two groups (IVIg within or after 48 h) was 23.3% (within 48 h) and 57.1% (after 48 h), indicating that early initiation of high-dose IVIg therapy may be beneficial in severe COVID-19 patients. On the contrary, there were no significant effects of IVIg therapy on the survival of severe COVID-19 patients who developed ARDS in another study; here, the insufficient effects of IVIg therapy may potentially be a result of timing and dosage of IVIg administration (220).

In another study, a combination therapy of a high dose of IVIg (20 g/d) and corticosteroid (160 mg/d) succeeded in reversing the deteriorating condition of severe COVID-19 patients who had previously failed low-dose IVIg (10 g/d) and corticosteroid treatment (221). However, as this study investigated a combined therapy, it is not possible to estimate the exact effect of high IVIg doses in this group of patients.

The administration of IVIg therapy (25 g/d for 5 days) at the time of initiation of respiratory distress of severe COVID-19 patients may improve the prognosis (16). It is noted that only one of three patients received corticosteroids, and these observations suggest that high-dose IVIg therapy may be the main contributor to successful recovery from deteriorating conditions. However, more controlled trials of IVIg therapy are needed to provide strong evidence for its beneficial effects in COVID-19.

Based on these clinical observations, the administration of high-dose IVIg therapy at the appropriate point may be able to prevent disease progression and improve the prognosis of patients with severe COVID-19. Currently ongoing randomized controlled trials of high-dose IVIg therapy in severe COVID-19 patients are evaluating the benefit of IVIg compared to standard care (NCT04261426 and NCT04350580) and will be able to further provide more information about the clinical effects of IVIg therapy in COVID-19.

Although a large number of clinical trials have demonstrated that IVIg therapy is well-tolerated, various side effects have been reported (244). The majority of these effects are mild and transient, yet clinicians need to be vigilant about rare but serious adverse effects such as aseptic meningitis, renal impairment, thrombosis, and hemolytic anemia (244). It is noted that thrombosis is common in COVID-19, and whether high-dose IVIg therapy would further increase the risk of thrombosis remains unclear (17, 245). However, the combined treatment of low molecular weight heparin and high-dose IVIg therapy at 0.3–0.5 g/kg/d for 5 days in the early phase has been suggested to have proper efficacy in treating severe COVID-19 patients (17).

## Conclusion

The IVIg preparation is a widely used pooled human blood product that can provide passive immunity and modulate the immune functions. Although, for COIVD-19 the pathogen-specific effects of IVIg are not relevant yet, since available preparations were collected from healthy donors without pre-existing immunity early before the pandemic began. The anti-inflammatory and immunomodulatory effects on the various immune cells of high-dose IVIg may account for its clinical benefits. Based on these potential supportive F(ab)′_2_ and Fc mediated mechanisms and the known clinical effects in treating severe virus pneumonia such as SARS, MERS, influenza, and RSV disease, the early application of high-dose IVIg therapy may be considered in the management of severe COVID-19 patients. Currently, limited clinical practice of high-dose IVIg in treating SARS-CoV-2 infection has been reported to show potential clinical benefits. Still, more research is needed but these inferences may provide relevant and useful insights and help in confronting the COVID-19 epidemic.

## Author Contributions

XL conceived and wrote the manuscript and prepared figures. WC and TL contributed to the modification and revision of the manuscript. All authors contributed to the article and approved the submitted version.

## Conflict of Interest

The authors declare that the research was conducted in the absence of any commercial or financial relationships that could be construed as a potential conflict of interest.
